# Differential Replication and Oncolytic Effects of Zika Virus in Aggressive CNS Tumor Cells: Insights from Organoid and Tumoroid Models

**DOI:** 10.3390/v16111764

**Published:** 2024-11-12

**Authors:** Rodolfo Sanches Ferreira, Elisa Helena Farias Jandrey, Isabela Granha, Alice Kei Endo, Raiane Oliveira Ferreira, Bruno Henrique Silva Araujo, Mayana Zatz, Oswaldo Keith Okamoto

**Affiliations:** 1 Human Genome and Stem Cell Research Center (CEGH-CEL), Department of Genetics and Evolutionary Biology, Institute of Biosciences, University of São Paulo, Cidade Universitária, São Paulo 05508-090, SP, Brazil; rodolfo.sanchesf@gmail.com (R.S.F.); elisajandrey@gmail.com (E.H.F.J.); isabelagranha@usp.br (I.G.); kei.endo30@gmail.com (A.K.E.); raianeferreira@ib.usp.br (R.O.F.); mayazatz@usp.br (M.Z.); 2 Brazilian Biosciences National Laboratory (LNBio), Brazilian Center for Research in Energy and Materials (CNPEM), Rua Giuseppe Máximo Scolfaro, No. 10.000, Campinas 13083-970, SP, Brazil; bhsa83@gmail.com; 3 Hemotherapy and Cellular Therapy Department, Hospital Israelita Albert Einstein, São Paulo 05652-900, SP, Brazil

**Keywords:** cancer, Zika virus, brain organoids, central nervous system tumors, glioblastoma, medulloblastoma, atypical teratoid rhabdoid tumor, virotherapy, oncolytic virus

## Abstract

Central nervous system (CNS) cancers are responsible for high rates of morbidity and mortality worldwide. Malignant CNS tumors such as adult Glioblastoma (GBM) and pediatric embryonal CNS tumors such as medulloblastoma (MED) and atypical teratoid rhabdoid tumors (ATRT) present relevant therapeutic challenges due to the lack of response to classic treatment regimens with radio and chemotherapy. Recent findings on the Zika virus’ (ZIKV) ability to infect and kill CNS neoplastic cells draw attention to the virus’ oncolytic potential. Studies demonstrating the safety of using ZIKV for treating malignant CNS tumors, enabling the translation of this approach to clinical trials, are scarce in the literature. Here we developed a co-culture model of mature human cerebral organoids assembled with GBM, MED or ATRT tumor cells and used these assembloids to test ZIKV oncolytic effect, replication potential and preferential targeting between normal and cancer cells. Our hybrid co-culture models allowed the tracking of tumor cell growth and invasion in cerebral organoids. ZIKV replication and ensuing accumulation in the culture medium was higher in organoids co-cultured with tumor cells than in isolated control organoids without tumor cells. ZIKV infection led to a significant reduction in tumor cell proportion in organoids with GBM and MED cells, but not with ATRT. Tumoroids (3D cultures of tumor cells alone) were efficiently infected by ZIKV. Interestingly, ZIKV rapidly replicated in GBM, MED, and ATRT tumoroids reaching significantly higher viral RNA accumulation levels than co-cultures. Moreover, ZIKV infection reduced viable cells number in MED and ATRT tumoroids but not in GBM tumoroids. Altogether, our findings indicate that ZIKV has greater replication rates in aggressive CNS tumor cells than in normal human cells comprising cerebral organoids. However, such higher ZIKV replication in tumor cells does not necessarily parallels oncolytic effects, suggesting cellular intrinsic and extrinsic factors mediating tumor cell death by ZIKV.

## 1. Introduction

Malignant tumors of the central nervous system (CNS) are among the deadliest forms of cancer, with both adult and pediatric patients facing poor prognoses. Glioblastoma (GBM) stands as the most aggressive and common primary CNS tumor in adults, while in pediatric patients, embryonal brain tumors such as medulloblastoma (MED) and atypical teratoid rhabdoid tumors (ATRT) also exhibit significant therapeutic challenges [1,2]. The overall survival rates for these patients are dismal and have been unaltered for decades due to minor responses to classical treatment regimens based on surgery, radio and/or chemotherapy [3], highlighting the urgent need for novel therapeutic approaches.

Oncolytic viruses are viral agents capable of infecting and killing cancer cells [4]. Recently, our group and others have reported Zika virus (ZIKV) to selectively infect and cause cell death in multiple CNS tumor types, including GBM, MED, and ATRT [5,6,7,8]. ZIKV shows a natural tropism to infect neural progenitor cells (NPC) and neural stem cells (NSC), reducing the cell population of the intra-utero developing brain and causing microcephaly [9,10,11]. Similarly to normal dedifferentiated brain cells, ZIKV preferably infects NPC-like and stem-like cancer cells [5,8]. Cancer stem cells (CSC) are a specific population of tumor cells with high self-renewal and tumor initiation capabilities; these cells are often referred to as responsible for treatment resistance and tumor recurrence [12]. ZIKV has been reported to selectively infect glioma stem cells (GSCs), both in vitro and in vivo, preferentially targeting SOX2+ cells across various CNS tumor types, including diffuse midline glioma, ependymoma, meningioma, MED, ATRT and GBM [13].

Studying CNS tumors in human-relevant models is essential for developing new treatment strategies. In this context, brain organoids generated from normal and healthy stem cells have emerged as an important tool for modeling the CNS tumor microenvironment in vitro. The co-culture of CNS tumor cells with brain organoids derived from human pluripotent stem cells provides an in vitro system that closely mimics in vivo tumor behavior, where cancer cells naturally infiltrate brain organoid tissue [14,15,16]. The co-culturing of brain organoids with CNS tumor cells has been used to investigate various novel therapies, including oncolytic viruses. Initial findings suggest that ZIKV preferentially infects and kills tumor cells from GBM, MED and ATRT, while showing limited effects on brain organoid cells [6,8]. Even at low multiplicity of infection (MOI), ZIKV’s oncolytic effects were also demonstrated in embryonal CNS tumor cell lines and tumorspheres [5]. However, virus replication, oncolytic potential and preference between normal and different cancer cells in humanized systems remain elusive. Understanding ZIKV differential behavior in human healthy and tumor tissues is important to clarifying the viral safety profile and potential applications in CNS tumor treatment.

Here we developed a co-culture model of human brain organoids with cancer cells from different CNS tumors and further used these assembloids to study ZIKV oncolytic effects. Our hybrid organoid–CNS tumor model enabled real-time tracking of tumor mass formation and growth in vitro. Using this model, we assessed ZIKV infection, its oncolytic effects, and viral replication in both tumor and non-tumor cells. Our findings reveal that ZIKV replicates at significantly higher rates in neoplastic CNS cells compared to normal neural organoid cells, supporting further clinical studies to evaluate the safety and tolerability of ZIKV as a potential oncolytic agent for treating CNS tumors.

## 2. Material and Methods

### 2.1. Cell Lines and Cultures

The previously established human induced pluripotent stem cell (hiPSC) line F9048 [17] and the human embryonic stem cell (hESC) line BR-6 [18] were cultured in mTeSR™ 1 (StemCell Technologies, Vancouver, BC, Canada) media with Normocin (100 µg/mL, Invivogen, San Diego, CA, USA) in Matrigel (hESC-qualified, Corning, Corning, NY, USA) coated 60 mm Petri dishes (Corning, NY USA). We obtained the human GBM cell line LN-18 (CRL-2610, ATCC, Manassas, VA, USA) and the Vero (CCL-81, ATCC) and HEK-293 (CRL-1573, ATCC) cell lines from the Human Genome and Stem Cell Studies Center (CEGH-CEL) cell biobank. The U343-MG (CVCL_S471) GBM cell line was kindly provided by Dr. Carlos Frederico Martins Menck’s research group. The human medulloblastoma cell line USP13-Med [19] and the atypical teratoid–rhabdoid tumor cell line USP7-ATRT [5] were established and characterized in-house. All tumor cell lines were grown in DMEM (Gibco, Waltham, MA, USA) medium supplemented with 10% fetal bovine serum (FBS, Gibco) and 1% Anti-Anti (Gibco, MA, USA). Cell culture was periodically tested for mycoplasma contamination using the Mycoalert Plus kit (Lonza, Basel, Switzerland). Standard culture conditions (5% CO_2_, 37 °C) were applied throughout all cell cultures.

### 2.2. Transduction of Tumor Cell Lines

Tumor cell lines used for co-culture and tumoroid generation were previously edited to express a Green Fluorescent Protein (GFP) reporter. Plasmids pMXs-IRES-GFP (RTV-013, Cell Biolabs, San Diego, CA, USA), psPAX2 (#12260, Addgene, Watertown, MA, USA) packaging vector and pMD2.G (#12259, Addgene) envelope vector, all bearing ampicillin resistance, were cloned into *E. coli* DH5α using LB-Broth, extracted (Plasmid Midi Kit, Qiagen, Hilden, Germany), quantified (Nanodrop™, Thermo Fisher Scientific, Waltham, MA, USA) and stored. For lentivirus production, HEK-293 cells were transfected with 4 µg of pMXs-IRES-GFP, 2 µg of psPAX-2, and 1 µg of pMD2.G using Lipofectamine 2000 (Thermo Fisher Scientific). The media containing viral particles was collected and filtered with a 0.45 µm filter after 48 h of transfection. All CNS tumor cell lines were seeded at 50% confluency and transduced with 1 mL of HEK viral supernatant for 24 h. Cells were expanded and selected twice through fluorescence-activated cell sorting (FACS, FACSAria™ II BD Biosciences, Piscataway, NJ, USA), achieving >90% of GFP+ cells for all four lines.

### 2.3. Brain Organoid Culture

We used a previously established protocol [20] to generate the brain organoids (see detailed mediums information in Table S1). The hPSCs were transferred to ultra-low-attachment round-bottom 96-well plates (Corning) at 10,000 cells per well in mTeSR 1 with Normocin (100 µg/mL) and Y-27632 (20 µM, Tocris, Bristol, UK); this was considered day 0 of organoid development. After 24 h, Y-27632-free mTeSR 1 medium was added to each embryoid body (EB). Media was gradually changed to HeSC media with 10 µM Y-27632 and 4 ng/mL bFGF (Peprotech, Waltham, MA, USA). After reaching 400–600 µm, EBs were transferred to ultra-low-attachment 24-well plates in N2 medium. After neuroepithelium cues appeared, EBs were embedded in Matrigel (Bemis, Sheboygan Falls, WI, USA) and then polymerized for 30 min at 37 °C. The droplets were transferred into ultra-low-attachment 6-well plates containing ND medium without vitamin A (6 to 10 organoids per well). The organoid plates were kept at 37 °C and 5% CO_2_ in static culture for four days, when the medium was changed to ND medium with vitamin A, and the plates were transferred to a shaker incubator at 65 rpm. The culture medium was changed every 3–4 days. After 40+ days of culture, organoids were considered mature and suitable for subsequent experiments.

### 2.4. CNS Tumor Cells Co-Culture with Brain Organoids and 3D Tumoroid Generation 

For co-culture assays, mature brain organoids (+40 days) were individually transferred to sterile 1.5 mL tubes in ND medium with vitamin A. Tumor cells were washed with PBS, released with Tryple, anf then centrifuged and resuspended in tumor cell culture medium. After counting, 100,000 GFP+ tumor cells were added to each organoid-containing tube. After 24 h of incubation, fresh ND media was added to each tube. The co-cultures were kept for another 24 h in 1.5 mL tubes to promote maximum tumor cell adherence to organoids. The resulting co-cultured models were then transferred to ultra-low-attachment 6-well plates (2 days post-co-culture) and kept in ND media with vitamin A. Co-cultured and control plates were maintained in a shaker incubator at 65 rpm until the viral infection assays. Media was changed every 3–4 days.

Tumoroids were developed by culturing CNS tumor cell lines in brain organoid maturation media. Briefly, tumor cell lines were washed with PBS, released with Tryple, centrifuged, and resuspended in ND media with vitamin A. Cells were plated in ultra-low-attachment 24-well plates at 100,000 cells per well. Fresh media was added on days 3 and 7 post-plating. After one week, tumoroids were considered ready for viral infection assays, and media was changed every 2 days.

### 2.5. In Vitro ZIKV Infection

The wild-type ZIKV17 strain (GenBank accession number ID: KU497555.1) was isolated from human testicular tissue and is described in Calvet et al., 2016 [21]. For the viral infection, co-cultured and control organoids were individually transferred to conical tubes, and excess media was removed. The viral suspension was prepared at 2 × 10^5^ PFU/mL in ND media, and 100 µL of pure media (mock groups) or 20,000 PFU ZIKV (ZIKV groups) was added to each tube. Viral infection was carried out for 2 h at 37 °C. Cell media was removed, and fresh ND media was added. The organoids were then transferred back to ultra-low-attachment 6-well plates. Similarly, for tumoroid infection, a 2 × 10^5^ PFU/mL ZIKV suspension was prepared. After 90% media was removed from each tumoroid-containing well, 100 µL of pure (mock groups) or 20,000 PFU ZIKV (ZIKV groups) ND media was added to each well. Viral infection was carried out for 2 h at 37 °C. After this period, the medium was removed, and fresh ND media was added. Both mock and ZIKV groups were maintained at 37 °C and 5% CO_2_ in static culture.

### 2.6. ZIKV Titration and Detection

Vero cells were seeded at 10^5^ cells/well in 24-well plates in DMEM high-glucose medium (2% FBS, 1% Anti-Anti) 24 h before the Plaque Forming Unit (PFU) assay. On the assay day, supernatants from infected and mock cultures were diluted 1:10 in DMEM high-glucose medium without supplements. Vero cell medium was replaced, and the supernatants of ZIKV-infected organoids were serially diluted (10-fold) in duplicate wells. The inoculum was incubated for 1 h at 37 °C and 5% CO_2_. A 0.6% Agarose solution (1:1 in DMEM high glucose) was added at 1 mL/well. After 4 days, the Agarose-containing medium was removed, and 4% formaldehyde (Sigma, MA, USA) was added for 20 min. After fixation, cells were stained with Crystal Violet (Sigma) for 15 min. Wells allowing the counting of the highest number of lyse plaques were selected. The PFU/mL was calculated using the formula [22]

PFU/mL=(average plaque count)/(Dilution×Volume of diluted sample)

ZIKV RNA presence in the culture supernatants was assessed via RT-qPCR. Viral RNA was extracted from supernatants using the QIAmp Viral RNA Mini Kit (Qiagen, Hilden, Germany). We used TaqMan Fast Virus 1-Step Master Mix (Thermo Fisher) with primers targeting a conserved viral genome region (see Table S2 for oligos details). Synthetic viral genome fragments (gBlock) were used as positive controls. A standard curve with known concentrations was generated and sample RNA was calculated based on Ct values, considering the volumes used for RNA extraction and RT-qPCR reaction.

### 2.7. Organoid Dissociation and Flow Cytometry

The cellular composition of co-cultured organoids was assessed through mechanical dissociation using cell strainers (Test Tube with Cell Strainer Snap Cap, Corning). Individual organoids were transferred to tube caps and gently pressed against the cell strainer mesh multiple times using a sterile syringe plunger. The strainer surface was washed multiple times with 1X PBS to maximize cell passage into the tube. The resulting cell suspension underwent centrifugation at 400× *g* for 5 min and was resuspended in either a fixative solution (4% formaldehyde) or PBS, depending on the assay. Following a 15 min fixation at room temperature, the cells were centrifuged and resuspended in PBS. Samples were promptly analyzed by flow cytometry or stored at 4 °C. All samples were analyzed in a FACSAria™ II BD Biosciences (NY, USA) flow cytometer.

### 2.8. Organoid Sectioning and Immunofluorescence Assays

For histological analysis of cerebral organoids, the samples were fixed with 4% formaldehyde for 24 h at 4 °C, cryoprotected in 15% sucrose solution for 4 h, followed by 30% sucrose for 24 h, all at 4 °C. The organoids were subsequently embedded in a 15% sucrose gelatin solution (Gelatin from porcine skin; Sigma-Aldrich). Embedded organoids were quickly frozen using a dry ice–ethanol bath (Merck Millipore, Darmstadt, Germany). The frozen samples were stored at −80 °C until sectioning. To obtain the tissue slices, organoids were equilibrated to the cutting temperature (−24 to −28 °C) for 15 to 20 min in a Microm HM 505E cryostat (Microm/Zeiss, Jena, Thuringia, Germany). Sections were acquired at 16–20 μm thickness, with 6 to 10 sections per slide and at least 5 slides per organoid.

For immunofluorescence (IF) staining, organoid slides were permeabilized with 0.3% Triton X-100 in 1X PBS for 20 min, blocked with 5% Bovine Serum Albumin (BSA, Sigma) in 1X PBS for 2 to 3 h, followed by incubation with the primary antibody for 1 h at room temperature or overnight at 4 °C (according to the manufacturer’s instructions for each antibody). Next, the slides were incubated with the secondary antibody for 1 h at room temperature (see detailed antibody information in Table S3). Between each step, the slides were washed three times with 1X PBS. After the final wash, ProLong™ Diamond Antifade Mountant with DAPI (ThermoFisher Scientific, Waltham, MA, USA) was applied, incubated for 5 min, and a coverslip was placed. Once dry, the slides were sealed with nail polish. The slides were immediately analyzed using a confocal microscope or stored at −20 °C until imaging.

hiPSCs and hESC were cultured on 8-well Lab-Tek^®^ Chamber Slides™ (Nunc^®^, Waltham, MA, USA). When colonies reached an adequate size, cells were fixed with 4% formaldehyde for 15 min at room temperature and washed with PBS. Cells were permeabilized and blocked (0.3% Triton X-100; 2% BSA) for 2 h at room temperature, followed by incubation with primary antibodies overnight at 4 °C (see detailed antibody information in Table S3). Next, the slides were incubated with the secondary antibody for 1 h at room temperature. Between each step, the slides were washed three times with 1X PBS. After the final wash, ProLong™ Diamond Antifade Mountant with DAPI (Thermo Fisher, MA USA) was applied, incubated for 5 min, and a coverslip was placed. Once dry, the slides were sealed with nail polish. The slides were immediately analyzed using a confocal microscope or stored at −20 °C until imaging.

For tumoroids IF stainings, whole tumoroids were fixed with 4% formaldehyde for 24 h at 4 °C, permeabilized with 0.3% Triton X-100 in 1X PBS for 30 min, and blocked with 3% BSA in 1X PBS for 2 to 3 h, followed by incubation with the primary antibody overnight at 4 °C. After washing with PBS, tumoroids were incubated with the secondary antibody for 2 h at room temperature, and then DAPI (2 µg/mL, Thermo Fisher) was added for 20 min at room temperature. Tumoroids were imaged whole using the z-stack tool.

### 2.9. Tumoroid Dissociation and Cell Viability Assays

CNS tumoroid models were dissociated with Accumax (StemCell Technologies, Vancouver, BC, Canada) for 15 min at 37 °C, homogenizing every 5 min. The cell suspension was passed through a 70 µm strainer (Corning), centrifuged and resuspended in tumoroid media. The CellTiter-Glo^®^ Luminescent Cell Viability Assay (Promega, Madison, WI, USA) was used to determine cell viability, following the manufacturer’s instructions. This viability essay is based on the quantitation of ATP, which is directly correlated to the amount of metabolically active cells. After incubation with the working reagent, the Relative Light Unit (RLU) of each sample was detected on a Lucetta^®^ 2 Luminometer (Lonza, Basel, Switzerland). Hence, the RLU measurement is proportional to the amount of viable cells.

### 2.10. Statistical Analysis and Imaging

Comparisons were conducted using Student *t*-test and two-way ANOVA, followed by Tukey’s post-test, in GraphPad Prism software (version 7.04). Live tumor-bearing organoid images were acquired on an AXIO (Zeiss, Thuringia, Germany) fluorescence microscope with ZEN Blue software (Zeiss, version 3.4). The Z-stack imaging of entire organoids, tumoroids and immunofluorescence slide imaging were performed on a confocal microscope AX 10 Observer.Z1 (Zeiss), also using ZEN Blue software. Brightfield images of tumoroids were acquired on an EVOS Digital Microscope (Life Technologies, Carlsbad, CA, USA) and area measurement was performed in ImageJ (Fiji, version 1.52p).

## 3. Results

### 3.1. CNS Tumor Cell Lines Attach and Spread Through Brain Organoids

Firstly, F9048 hiPSCs used for organoid development were checked for pluripotency markers expression and genomic integrity (Figure S1). As expected, F9048 hiPSCs cells expressed the pluripotency protein markers NANOG, OCT4A, SOX2 and SSEA-4 (Figure S1A). We also observed similar levels of *NANOG* and *OCT4A* expression when comparing trasncript levels in the F9048 hiPSCs and the hESC line BR-6 (Figure S1B). The F9048 hiPSCs presented genomic integrity, with the absence of detected discrepancies in CGH-array and MLPA assays (Figure S1C). These data confirm the suitability of using F9048 hiPSCs for modeling brain organoids.

To obtain the hybrid co-culture model, brain organoids were first developed and characterized. After 40 days, our cerebral organoids displayed organized neuroepithelium and pseudo-ventricular cavities (Figure S2A). Structures similar to the ventricular zone (VZ) populated by inner layer SOX2+ radial glial cells were observed, as well as outer radial glial SOX2+ cells (Figure S2B). Doublecortin (DCX)+ neurons/pre-neurons were organized in a structure resembling the cortical plate, surrounding the VZ and pseudo-ventricular cavities (Figure S2B). The immunofluorescence of 90-day-old organoids revealed TUJ1/β-Tubulin III+ neurons, in an epithelium resembling the cortical plate, surrounded by diffuse populations of SOX2+ cells (Figure S2C). As expected, our brain organoids presented tissue architectures and compositions similar to the “whole brain organoids” developed in previous studies [20].

**Figure 1:**
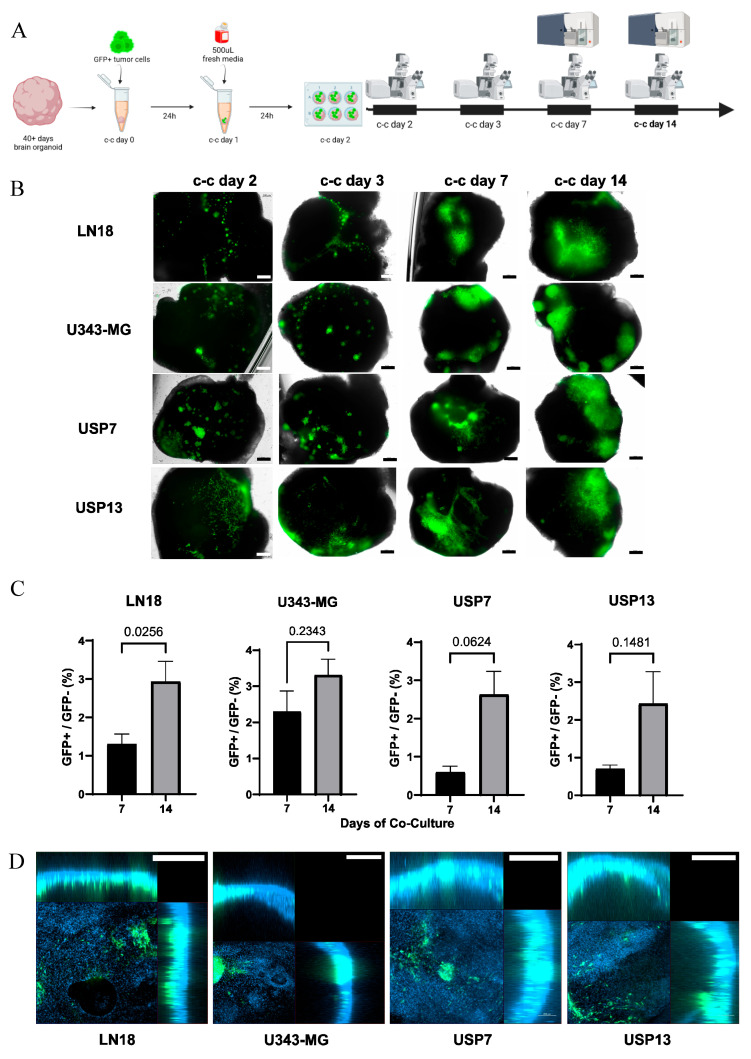
Co-culture of brain organoids and CNS tumor cell lines. (**A**) Schematic representation of co-culture assay and analyses timeline. (**B**) Brightfield-fluorescence images of co-cultured brain organoids and GFP+ LN18 GBM, U343-MG GBM, USP7 ATRT and USP13 MED cell lines (scale bar 200 µm) (c-c: co-culture). (**C**) Flow cytometry quantification of GFP+/GFP− (tumor/non-tumor) cell proportion (%) in co-cultured groups, *n* = 3. (**D**) Orthogonal view images from c-c day 14 whole co-cultured organoids (scale bar 100 µm).

We generated hybrid co-culture models of brain organoids with four GFP+ CNS tumor cell lines, two of them representing glioblastoma (LN18 and U343-MG), and the other two representing embryonal CNS tumors (USP7-ATRT and USP13-Med). A total of 10^5^ tumor cells were added per organoid. Organoids without tumor cell addition were used as control groups. Brightfield-fluorescence images were acquired on co-culture (c-c) days 2, 3, 7 and 14, and cell composition was assessed on c-c days 7 and 14 (Figure 1A). We observed variable amounts of GFP+ tumor cells attached to the neural tissue at c-c day 2, and signs of tissue invasion and different migration patterns between the cell lines were observed at c-c day 3 (Figure 1B). Particularly, but not exclusively, the U343-MG group displayed tumor-like structures with a dense cellular mass and invasive protrusions. Similar structures were also detected in the USP7 group at c-c day 14, while the USP13 cell line showed abundant cell migration at this timepoint (Figure 1B). Independent of the tumor dispersion pattern into the brain organoids, these data show the capability of the LN18, U343-MG, USP7 and USP13 cells to adhere and grow in this organoid model.

Tissue composition analysis at c-c days 7 and 14 showed an increase in GFP+/GFP− (tumor/non-tumor) cell proportion over time for all cell lines but, due to inter-organoid variability, this difference was statistically significant only for LN18 (Figure 1C). The LN18 GBM showed the most pronounced increase, rising from an average of 3% at 7 days post-c-c to 9.1% at 14 days post-c-c, followed by USP7 (0.6% to 2.6%), USP13 (0.7% to 2.4%), and U343-MG (2.3% to 3.3%). Our co-culture system generated hybrid organoids–CNS tumor models with varying amounts of adhered GFP+ cells. Despite the different dynamics of tissue occupation, all co-cultured tumor cell lines increased their proportion over non-tumor GFP cells over time.

To determine if tumor cells remained on the surface or invaded the organoid tissue, we nuclei-stained and z-stack-imaged whole 14-day co-cultured models in a confocal microscope. Maximum projection orthogonal view images revealed that all four CNS tumor cell lines reached the inner regions of the organoids after two weeks (Figure 1D). These results demonstrate that our co-culture models provide a human neural multicellular platform permissive to tumor cell attachment, growth, and invasion, thus confirming cerebral organoid suitability for studying CNS tumors in vitro.

### 3.2. ZIKV Infection Decreased GBM and MED, but Not ATRT, Tumor Cell Proportion in Brain Organoids

**Figure 2:**
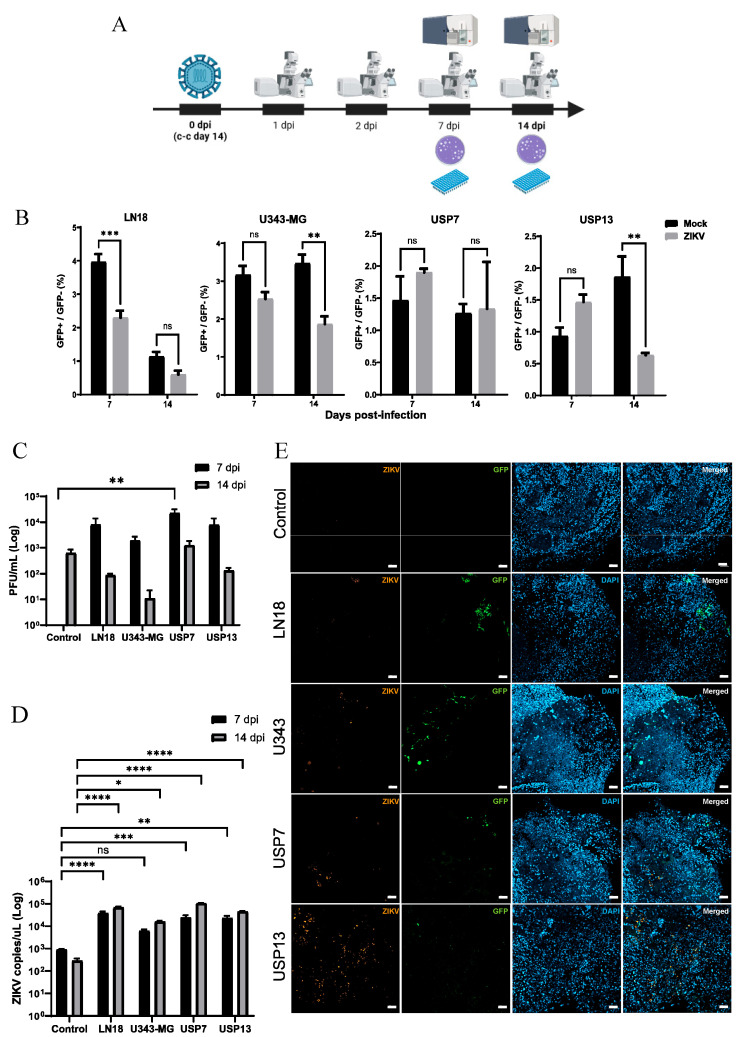
ZIKV infection of co-cultured and control organoids. (**A**) Schematic representation of viral infection assay and analyses timeline (dpi, days post-infection; c-c, co-culture). (**B**) Cell composition of mock and ZIKV-treated co-culture groups at 7- and 14-dpi, *n* = 3 (ns: non-significant). ** *p* < 0.01. *** *p* < 0.001. (**C**) Supernatants Plaque Forming Unit (PFU) concentration of ZIKV-treated co-cultured and control groups at 7- and 14-dpi. ** *p* < 0.01. (**D**) RT-qPCR viral RNA quantification of ZIKV-treated co-cultured and control groups at 7- and 14-dpi (ns: non-significant). * *p* < 0.05. ** *p* < 0.01. *** *p* < 0.001. **** *p* < 0.0001. (**E**) Immunofluorescence detection of non-structural ZIKVBR protein NS2B (orange) and GFP+ tumor cells (green) in 14-dpi control and co-cultured organoids (scale bar 50 µm).

Two-week control and co-cultured organoids were split into mock groups and ZIKV groups for the viral infection assays (Figure 2A). One and two days post-infection (1- and 2-dpi), no clear tumor GFP+ cell mass reduction was observed in ZIKV-treated groups (Figure S3).

The flow cytometry cell composition analysis of organoids revealed the LN18 ZIKV group showed a significant reduction (*p* < 0.001) in tumor cell proportion compared to the respective mock group (Figure 2B). The U343-MG ZIKV group had a lower proportion of GFP+ cells (2.53%) compared to the U343-MG mock group (3.16%), but this difference was not statistically significant. Interestingly, USP7 and USP13 had similar mock and ZIKV-treated tumor/non-tumor cell proportions at 7 dpi. At 14 dpi, all groups, except for USP7, showed a lower proportion of GFP+ cells in infected organoids compared to respective mocks (Figure 2B). This difference was significant for the U343-MG (*p* < 0.01) and USP13 (*p* < 0.01) groups. These results suggest a significant oncolytic effect of ZIKV against GBM and MED tumor cell lines, but not against the USP7 ATRT cell line, in our hybrid brain organoids–CNS tumor model.

### 3.3. ZIKV Has Greater Replication in the Presence of CNS Tumor Cells

To investigate ZIKV infection and replication in hybrid and control organoids, infectious viral particle quantification and RNA copies concentrations were assessed through plaque-forming units (PFU) and RT-qPCR assays, respectively. Culture supernatants were collected in triplicates and analyzed at 7 and 14 dpi (Figure 2A). In the tumor-free control groups, viral particles were only detected at 14 dpi (Figure 2C). In contrast, infectious viral particles were present in the supernatants of all infected co-cultured tumor cell lines, both at 7 and 14 dpi, and the concentrations were higher one week post-infection in all hybrid groups in comparison with control organoids (Figure 2C). Interestingly, USP7 presented the highest infectious viral load at both timepoints, being significantly higher than the control group at 7 dpi.

Viral RNA concentration increased in all co-cultured groups over time (7 vs. 14 dpi), while it decreased in the tumor-free control groups (Figure 2D). Except for U343-MG at 7 dpi, all co-cultured groups had significantly higher viral copy concentrations than controls in both timepoints (Figure 2D). The immunofluorescence staining of 14 dpi organoids confirmed ZIKV presence in all co-cultured groups, and positive ZIKV staining was found around GFP+ areas, while little to no ZIKV protein was detected in control organoids (Figure 2E).

Our data reveal a reduced viral infection and replication in isolated brain organoids, while the presence of GBM, ATRT, or MED tumor cells significantly augmented ZIKV viral replication in vitro. Interestingly, viral particle accumulation was augmented even in the cell line that showed no reduction in tumor cell proportion over time (USP7, Figure 2B), indicating a high replication rate not necessarily accompanied by oncolytic effect in ATRT cells in vitro.

### 3.4. ZIKV Infects and Rapidly Replicates in CNS Tumoroids

**Figure 3:**
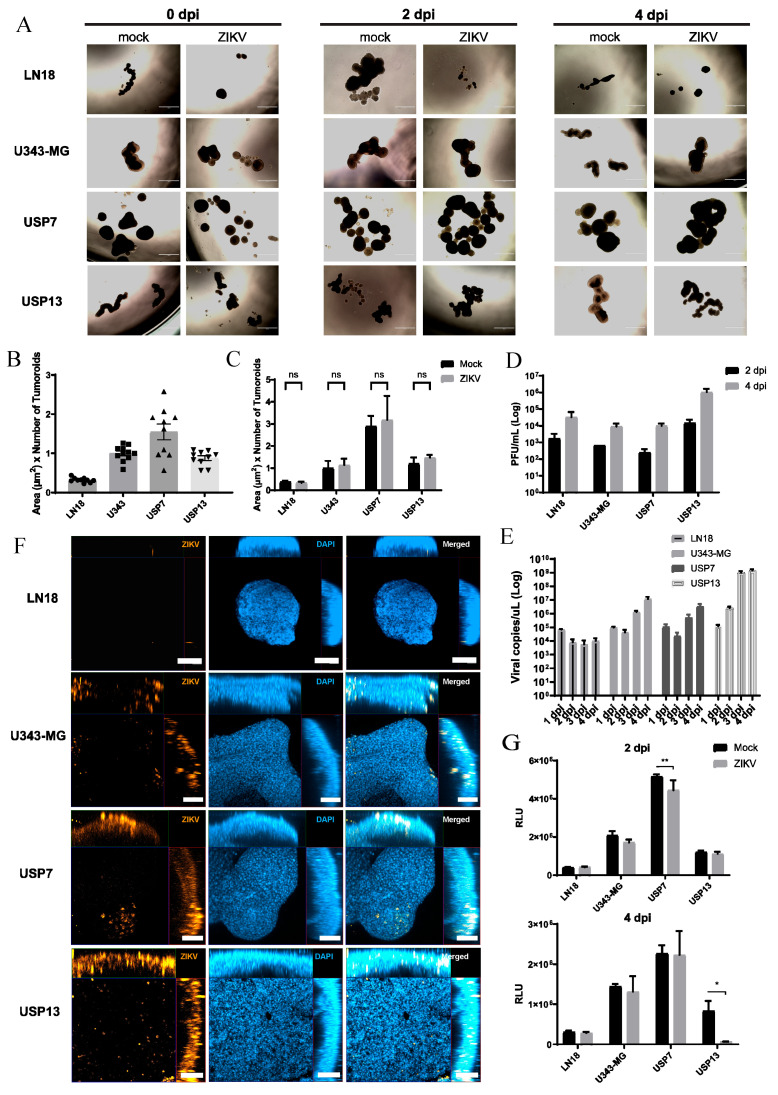
ZIKV infection effects on three-dimensional CNS tumoroids. (**A**) Brightfield images of tumoroids at 0, 2 and 4 dpi (scale bar 1000 µm). (**B**,**C**) Measurement of area (µm^2^) multiplied by the number of formed tumoroids (**B**) at 7 days (0 dpi) after plating and (**C**) at 4 dpi (ns: non-significant). (**D**) Supernatants PFU/mL concentration of ZIKV-treated tumoroids at 2 and 4 dpi. (**E**) the RT-qPCR viral RNA quantification of ZIKV-treated tumoroids supernatant at 2 and 4 dpi. (**F**) Orthogonal view images of whole tumoroids immunolabeled for ZIKV protein NS2B (orange) at 4 dpi (scale bar 50 µm). (**G**) Luminescence assay for tumoroids cell viability, where RLU measurement is proportional to live cell number, at 2 (above) and 4 dpi (below). * *p* < 0.05. ** *p* < 0.01.

To further investigate ZIKV replication in tumor cells, we generated tumor-like models by culturing CNS tumor cell lines in suspension on brain organoid maturation medium. We deemed these structures tumoroids. After 7 days of plating, the different tumor cell lines formed three-dimensional structures at variable sizes, formats, densities and numbers (Figure 3A). The two embryonal CNS tumor cell lines, USP7 and USP13, formed the largest and most numerous tumoroids, while the GBM cell line U343-MG generated fewer and denser structures (Figure 3B). The LN18 GBM formed the scarcest and smallest tumoroids among all lines (Figure 3B).

Tumoroids from each cell line were split into mock and ZIKV groups seven days after plating (0 dpi). ZIKV infection was carried out as with the co-culture models. Tumoroid area and number analysis of treated and mock groups at 4 dpi revealed no significant reduction in number and size upon ZIKV infection (Figure 3C).

PFU analysis of supernatants at 2 and 4 dpi confirmed an increase in infectious viral particle concentration over time in all treated groups (Figure 3D). Except for LN18, this increase was followed by an accumulation of high amounts of ZIKV genetic material in all groups (Figure 3E). Interestingly, viral RNA accumulation was higher in tumoroids of all groups when compared to the respective co-culture models, even in a shorter period (4 days against 14 days) (Figure 2C and Figure 3E). This suggests that ZIKV has a greater replication rate in CNS tumor cell lines than in human neural organoid cells in vitro, regardless of the tumor type (GBM, MED or ATRT). Immunofluorescence staining and z-stack imaging confirmed the presence of ZIKV-infected cells in tumoroids of all four groups (Figure 3F).

To determine if the viral infection and replication were accompanied by an oncolytic effect in the tumoroids, cell viability was assessed by luminometry assays at 2 and 4 dpi. Surprisingly, only USP7- and USP13-infected groups displayed lower cell viability, at 2 dpi and 4 dpi, respectively (Figure 3G). No significant reduction in viability was observed in GBM-infected groups at either of the timepoints. These results suggest that ZIKV infects and rapidly replicates in three-dimensionally cultured CNS tumor cells in vitro, independently of the presence of non-tumor cells, but an oncolytic effect does not necessarily parallel this viral accumulation. Cells present in the microenvironment of the tumor–brain organoid assembloid may help in modulating the tumor cell infection rates and death response in vitro.

## 4. Discussion and Conclusions

As interest in viral therapies for cancer treatment continues to grow, ZIKV has emerged as a promising candidate against CNS tumors, particularly pediatric brain tumors with neural progenitor origins. ZIKV’s distinctive abilities to cross the blood–brain barrier, selectively target stem-like cancer cells, and activate an anti-tumor immune response make it a valuable candidate for novel therapies. Brain tumors are notoriously difficult to treat, as their isolation behind the brain’s immune defenses and the presence of CSCs often lead to the failure of conventional treatments and newer immunotherapies [23].

Here, we show that the co-culture of brain organoids and CNS tumor cells provides a human in vitro model where cancer cells spontaneously invade brain organoid tissue. The treatment of our hybrid models with ZIKV led to a reduction in cancer cell proportion in two GBM cell lines, LN18 and U343-MG, and one MED cell line, USP13, while ATRT USP7 cell proportion was not affected by the viral treatment. This is the first work to demonstrate ZIKV infection of the LN18 GBM cell line. Previously published data on LN18 and U343-MG show that both cell lines have gene expression and mutation profiles associated with mesenchymal GBM [24,25], suggesting this subtype could be susceptible to ZIKV oncolytic effect. There is one case report of a GBM patient who, after the standard treatment, was clinically diagnosed with ZIKV during an outbreak in Brazil. Interestingly, after the infection’s resolution, the tumor regressed without recurrence so far [26]. Of note, ZIKV’s effects on GBM cells have mostly been reported on GSC-based cell models [7,8,14,27], while data on the virus affecting tumor cell lines and differentiated cancer cells are scarce.

The initial ZIKV CNS oncolytic studies were focused on embryonal CNS tumors [5]. Here, the USP7-ATRT cell line was the only infected group that showed no reduction in tumor/non-tumor cell proportion in either timepoint, and also the cell line with the highest infectious viral particle accumulation. Whole-genome sequencing, chromosomal aberration and gene expression profile analysis revealed that USP7’s molecular profile closely resembles that of NSCs, to which ZIKV has a high tropism. This phenotypic similarity could explain the high infection rate and viral replication in these cells [28,29]. Similarly, a previous study from our group using 26-day brain organoids co-cultured with USP7 showed that treatment with one or three doses of 2000 PFU ZIKV led to a high production of infectious viral particles that was not accompanied by a significant reduction in tumor mass [6]. However, these results conflict with those of a previous study wherein ZIKV significantly reduced USP7 mice tumors in vivo, and tumorspheres in vitro [5]. These apparently conflicting results could be partly caused by the differences in the experimental models. Our present and previous (2021) studies cultured the tumor cells in brain organoid maturation media, whose composition favors the growth and maintenance of neural stem and progenitor cells, as well as neurons [30]. Hypothetically, this environment can favor cellular growth at an accelerated pace, which would compensate for the oncolytic effect of ZIKV. This is also supported by the fact that USP7 generated the largest and most abundant tumoroids among all tumor cell lines in the present study. Notably, despite no significant reduction in USP7 cells grown in normal cerebral organoids, ZIKV treatment did reduce the amount of viable USP7 tumor cells grown alone as tumoroids, suggesting that interaction between tumor and normal cerebral cells may affect ZIKV’s oncolytic effects.

The cerebral organoid model used in this work presents some limitations worth addressing. The major one is the absence of an immunological component [31]. ZIKV has been shown to induce a pronounced anti-tumor immune response in vivo [32,33], and the depletion of specific lymphocytes abrogated ZIKV oncolytic effects in murine glioma models [32,34]. Considering the characteristics of oncolytic viruses to induce a relevant anti-tumoral immune response, and that this response plays a role in the viral anti-tumoral effect [35,36], the absence of immune cells could also explain the viral replication not necessarily causing a reduction in tumor cell proportion in our co-culture model. In this sense, the lack of vascular cells in the cerebral organoids is another limitation worth mentioning in our model, given that vasculature is important for enabling immune cells and signaling molecules to interact with brain tissue, thereby contributing to the immune response against tumors.

ZIKV has been shown to infect, replicate and induce the cell death of NPCs and NSCs in brain organoids, causing reduced organoid size [29,37,38]. We detected a significant accumulation of ZIKV genetic material in organoids co-cultured with all four tumor cell lines in comparison with isolated controls. The viral selectivity between normal and tumor cells is an important factor regarding the ZIKV safety profile. Recently developed genetically modified and attenuated versions of ZIKV showed high selectivity and replication in tumor cells, while sparing normal cells [39,40]. Notably, our co-culture model applies to testing such modified novel agents.

The infection of in vitro human tumor models with ZIKV has been previously reported [5,7,8,41,42], whereby viral treatment impaired tumor cell growth and caused cell death in tumorspheres and GSCs models. The viral preference for progenitor-like cells has been established, with SOX2+ cells most likely to be infected [8]. Here, we have shown that ZIKV rapidly infects and replicates in CNS tumoroids, cancer cell models cultured in NPC/neuron maintenance media. The viral load increased in a short period of time, accumulating more viral genetic material than in co-cultured organoids. Even though this effect was not necessarily followed by a reduction in cell viability, this is notable considering mature brain organoids carry many progenitor SOX2+ cells, suggesting that ZIKV thrives in CNS tumor environments even compared to NPC-rich normal tissue.

Taken together, our data reveal different effects of ZIKV infection in cell lines representing three types of aggressive CNS tumors, showing increased viral replication on GBM, MED and ATRT cancer cells over normal cells, and reducing GBM and MED cell proportions in our human brain organoid–tumor co-culture models. Our findings emphasize ZIKV’s therapeutic potential, highlighting viral selectivity for replication in distinct aggressive CNS tumor cells. While these results provide a rationale for early clinical trials to evaluate safety, further investigations of the differential viral effects on healthy adult brain cells and neoplastic cells, especially in the presence of immune cells, are required to elucidate ZIKV’s full therapeutic potential.

## Data Availability

There are no additional data supporting the results reported in this study.

## Supplementary Materials

The following supporting information can be downloaded at: https://www.mdpi.com/article/10.3390/v16111764/s1, Figure S1: Pluripotency markers expression and genomic integrity of F9048. Figure S2: Brain organoid immunofluorescence tissue composition characterization. Figure S3: Brightfield-fluorescence images of assembloids at 1, 2, 7 and 14 dpi. Table S1: Media composition for brain organoids development. Table S2: Primers and oligos sequences. Table S3: Antibodies used for immunofluorescence.
