# Supplementary material for: Differential Replication and Oncolytic Effects of Zika Virus in Aggressive CNS Tumor Cells: Insights from Organoid and Tumoroid Models
Source: Viruses. 2024 Nov 12;16(11):1764. doi: 10.3390/v16111764 (PMC11598871; doi:10.3390/v16111764)
Supplement: Supplementary file 1 [file viruses-16-01764-s001.zip › viruses-3263267-supplementary.pdf]

Supplementary Materials

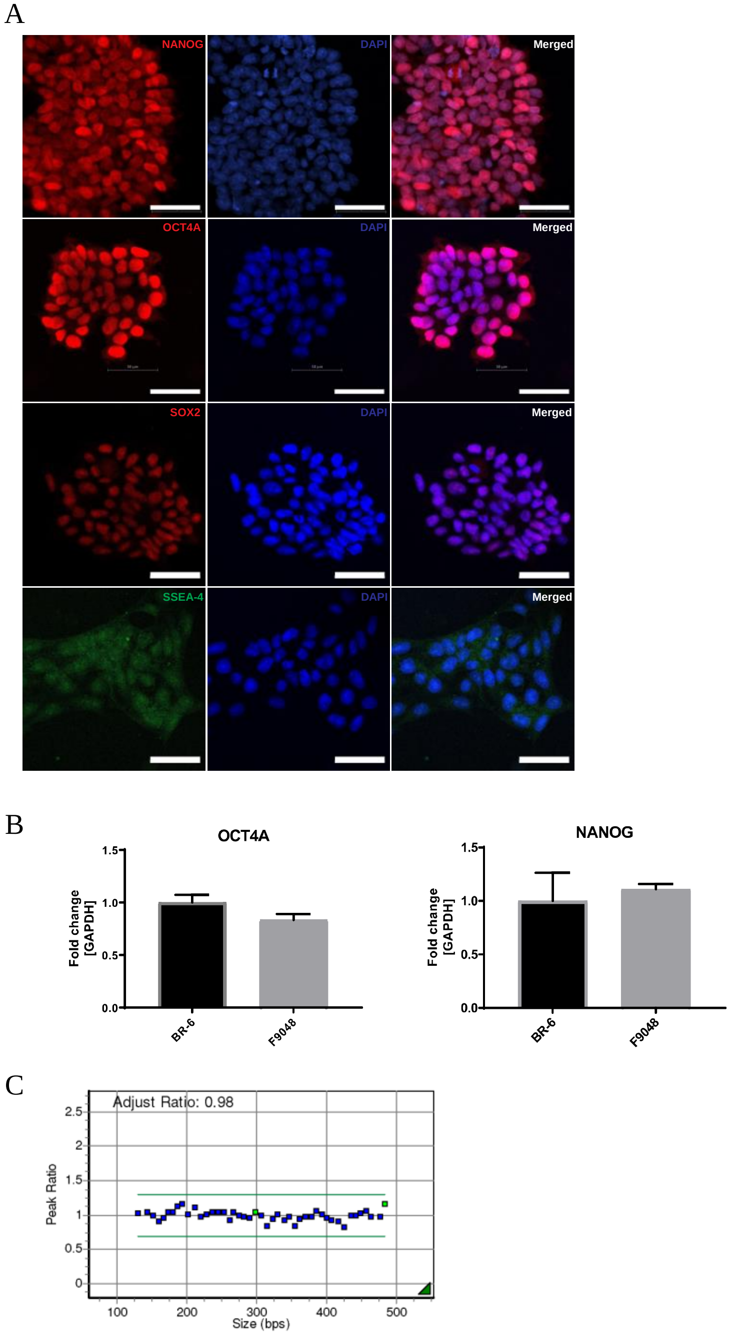

Supplemental Figure S1 – Pluripotency markers expression and genomic integrity of F9048. (A) Immunofluorescence staining for Nanog (red), Sox2 (red), OCT4A (red) and SSEA-4 (green) in F9048 colonies (scale bar 100 µm). (B) Relative mRNA expression of OCT4A (left) and NANOG (right) in F9048 compared to hESC BR6 (expression normalized to GAPDH). (C) Multiplex Ligation-dependent Probe Amplification (MLPA) analysis of F9048 genomic DNA. For each probe, the peak ratio <0.7 stands for deletion; and the ratio >1.3 stands for duplication.

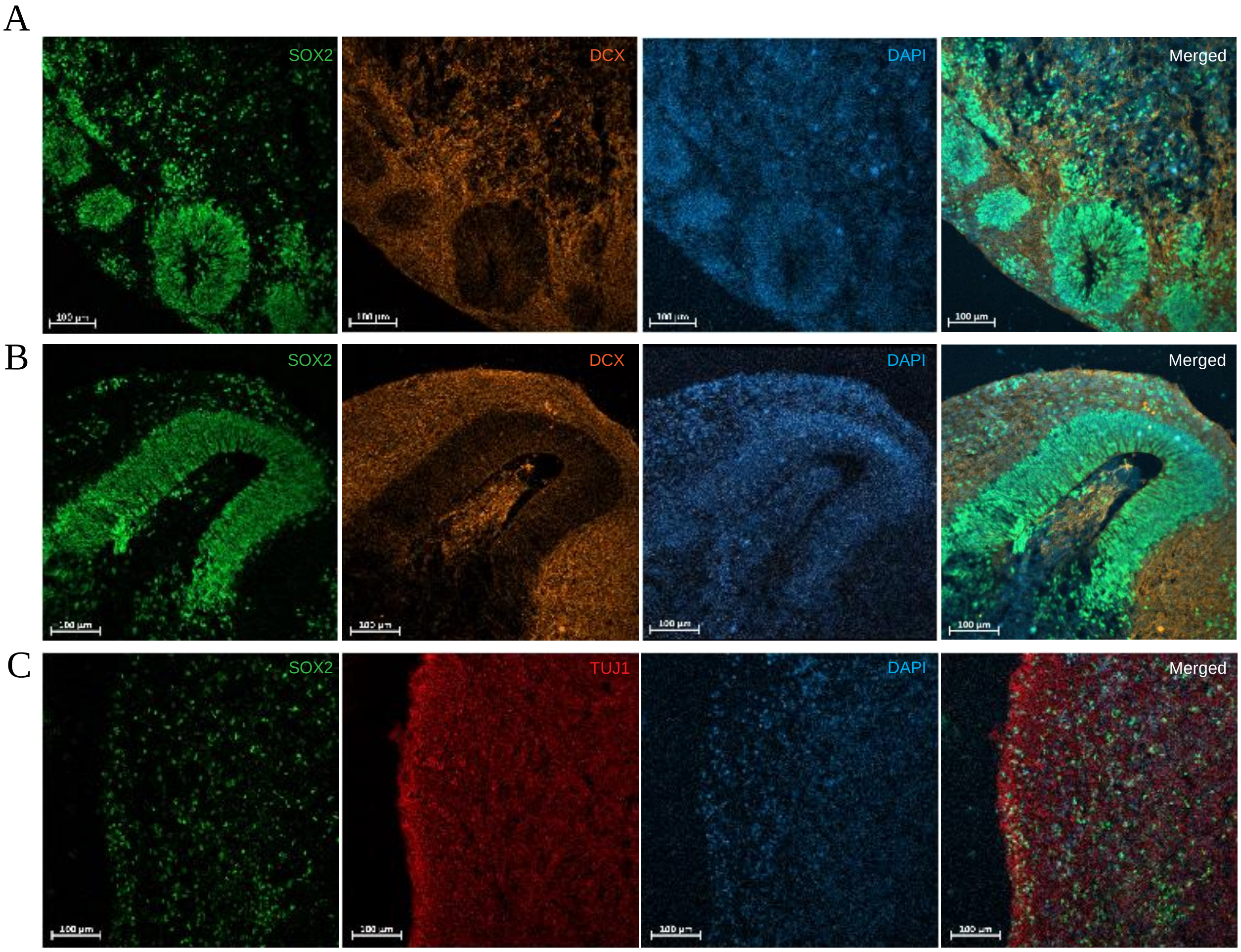

Supplemental Figure S2 – Brain organoid immunofluorescence tissue composition characterization. SOX2+ (green) neuroprogenitor cells and Doublecortin+ (DCX, orange) early neurons organization in 40-days brain organoids (A,B) and TUJ1 (red) (C) early neurons in 90-day organoids (scale bar 100 μm).

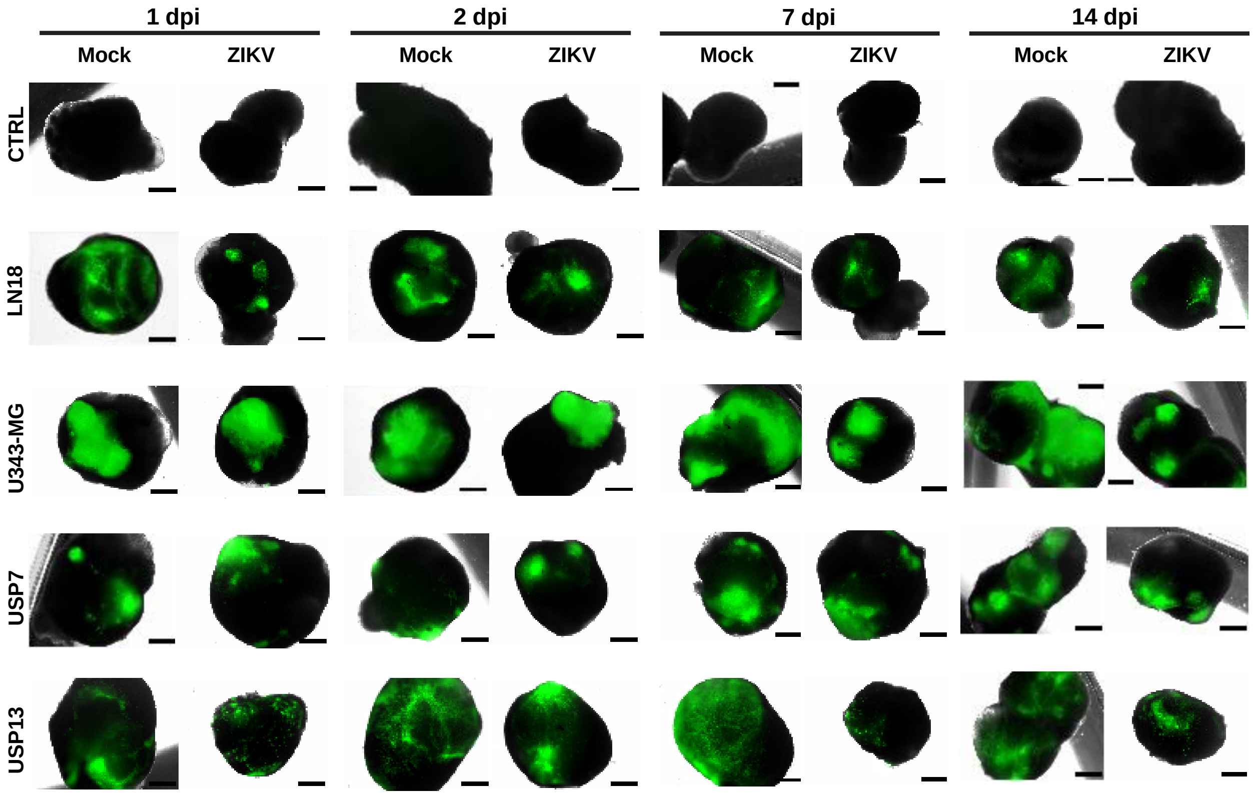

Supplemental Figure S3 – Brightfield-fluorescence images of assembloids (co-cultured organoids and GFP+ CNS tumor cells) and controls at 1, 2, 7 and 14 dpi (scale bar 200 μm).

Table S1 – Media composition for brain organoid development.

Media	Composition
HeSC	DMEM/F12 (Gibco)<br>20% Knockout Serum Replacement (KSR, Gibco)<br>1% MEM Non-Essential Amino Acid Solution (NEAAS, Gibco)<br>1% GlutaMAX (Gibco)<br>0.1 mM 2-mercaptoethanol (Gibco)
N2	DMEM/F12<br>1% N2 Supplement (Gibco)<br>1% MEM NEAAS<br>1µg/mL Heparin (Sigma Aldrich)
ND	DMEM/F12<br>Neurobasal (Gibco)<br>0.5% N2 supplement<br>1% B27 supplement without Vitamin A (Gibco) or<br>1% B27 supplement with Vitamin A (Gibco)<br>0.5% MEM NEAAS<br>1% GlutaMAX<br>50 µM 2-mercaptoethanol<br>2.5 µg/mL Human Insulin (Sigma Aldrich)<br>100 µg/mL Normocin

Table S2 - Primers and oligos sequences

**Primer / Oligo**	**Forward sequence**	**Reverse sequence**
OCT4A	5′-TCGCAAGCCCTCATTTCACCA-3′	5′-GGACTCCTCCGGGTTTTGCT-3
NANOG	5′-CCTGAAGAAAACTATCCATCC-3′	5′-CCTTGTCTTCCTTTTTTGCGA-3′
GAPDH	5′-GCATCCTGGGCTACACTG-3'	5′-CCACCACCCTGTTGCTGCTGTA-3′
ZIKV 1086 (F)	5′-CCGCTGCCCAACACAAG-3′	-
ZIKV 1162c (R)	-	5′-CCACTAACGTTCTTTTGCAGACAT-3′
ZIKV probe	5′-FAM AGCCTACCTTGACAAGCAGTCAGACACTCAA-3′ BHQ1	
ZIKV gBlocks® Gene Fragment	5′- GCATCAATATCAGACATGGCTTCGGACAGCCGCTG CCCAACACAAGGTGAAGCCTACCTTGACAAGCAAT CAGACACTCAATATGTCTGCAAAAGAACGTTAGTG GACAGAGGCTGGGGAAATGG-3′	

Table S3 - Antibodies used for immunofluorescence.

**Antibody**	**Catalog / Company**
Nanog	#4903 / Cell Signaling
Oct-4A	#2840C / Cell Signaling
Sox2	#3579 / Cell Signaling
Alexa Fluor® 488<br>anti-SSEA-4	560308 / BD Biosciences
Sox2	AF2018 / R&D Systems
Doublecortin	#48-1200 / Thermo Fisher Scientific
Tubulin, beta III (TUJ1)	MAB1637 / Merck
Zika virus NS2B protein	GTX133308 / GeneTex
anti-Rabbit Alexa Fluor 647	#A-21245 / Thermo Fisher Scientific
anti-Mouse Alexa Fluor 555	#A-21422 / Thermo Fisher Scientific
anti-Rabbit Alexa Fluor 594	111-585-144 / Jackson ImmunoResearch
anti-Goat Alexa Fluor Plus 488	#A32814 / Thermo Fisher Scientific
anti-Rabbit Alexa Fluor 555	#A31572 / Thermo Fisher Scientific
anti-Mouse Alexa Fluor 488	#A11029 / Thermo Fisher Scientific
